# Ecogenomics and Adaptation Strategies of Southern Ocean Viral Communities

**DOI:** 10.1128/mSystems.00396-21

**Published:** 2021-08-10

**Authors:** Tomás Alarcón-Schumacher, Sergio Guajardo-Leiva, Manuel Martinez-Garcia, Beatriz Díez

**Affiliations:** a Department of Molecular Genetics and Microbiology, Pontificia Universidad Católica de Chile, Santiago, Chile; b Max Planck Institute for Marine Microbiology, Bremen, Germany; c Department of Physiology, Genetics, and Microbiology, University of Alicante, Carretera San Vicente del Raspeig, San Vicente del Raspeig, Alicante, Spain; d Center for Climate and Resilience Research (CR) 2, Santiago, Chile; e Center for Genome Regulation (CGR), Santiago, Chile; UiT-The Arctic University of Norway

**Keywords:** viral diversity, Southern Ocean, molecular and physiological adaptations, stress adaptation

## Abstract

The Southern Ocean (SO) represents up to one-fifth of the total carbon drawdown worldwide. Intense selective pressures (low temperature, high UV radiation, and strong seasonality) and physical isolation characterize the SO, serving as a “natural” laboratory for the study of ecogenomics and unique adaptations of endemic viral populations. Here, we report 2,416 novel viral genomes from the SO, obtained from newly sequenced viral metagenomes in combination with mining of publicly available data sets, which represents a 25% increase in the SO viral genomes reported to date. They comprised 567 viral clusters (defined as approximately genus-level groups), with 186 genera endemic to the SO, demonstrating that the SO viral community is predominantly constituted by a large pool of genetically divergent viral species from widespread viral families. The predicted proteome from SO viruses revealed that several protein clusters related to cold-shock-event responses and quorum-sensing mechanisms involved in the lysogenic-lytic cycle shift decision were under positive selection, which is ultimately important for fine adaptation of viral populations in response to the strong selective pressures of the SO. Finally, changes in the hydrophobicity patterns and amino acid frequencies suggested marked temperature-driven genetic selection of the SO viral proteome. Our data provide valuable insights into how viruses adapt and remain successful in this extreme polar marine environment.

**IMPORTANCE** Viruses are the most abundant biologic entities in marine systems and strongly influence the microbial community composition and diversity. However, little is known about viral communities’ adaptation and diversification in the ocean. In this work, we take advantage of the geographical isolation and the intense selective pressures of the SO, to which viruses are exposed, to identify potential viral adaptations due to positive environmental selection and dispersal limitation. To that end, we recovered more than two thousand novel viral genomes, revealing a high degree of divergence in these SO endemic communities. Furthermore, we describe remarkable viral adaptations in amino acid frequencies and accessory proteins related to cold shock response and quorum sensing that allow them to thrive at lower temperatures. Consequently, our work greatly expands the understanding of the diversification of the viral communities of the SO and their particular adaptations to low temperatures.

## INTRODUCTION

The Southern Ocean (SO) is one of the most productive marine regions in the world. It plays a crucial role in carbon drawdown, contributing to an estimated 20% of the global oceanic CO_2_ uptake (1). In the SO, seasonality, and consequently the stratification of the water column, lead to the emergence of large phytoplankton blooms (2–4). In this scenario, the abundance of some specific bacterial taxa increases in response to specific phytoplankton bloom species (5). Therefore, bacterioplankton production is constrained by the phytoplankton species composition, bloom intensity, and duration. In turn, this microbial food web is regulated by viruses that alter the microbial community dynamics and functions (6–8). Viral infections can cause 3 to 68% of bacterial mortality in marine systems, thereby modifying biogeochemical cycles through the release of cellular contents during lysis (9–11). In this context, lytic replication dominates the viral community as the bacterial production increases during seasonal phytoplankton blooms. Positive feedback through viral-mediated nutrient release prompts the viral community to switch to a lysogenic cycle when bacterial production is low (12). Although the lytic-lysogenic switch mechanism is mostly unknown in Antarctic waters, the lysogenic cycle offers a plausible mechanism for viral prevalence under the harsh winter conditions (12).

Furthermore, the SO is considered more enclosed and isolated than other oceanic regions because its water masses are strongly linked by the circulation of the Antarctic Circumpolar current (13). Consequently, this allowed the development of endemic populations, as has been shown for different animals, plants (14, 15), and microbial communities in the maritime polar front boundaries when compared to other oceanic regions (16–18). Advection force is one of the most important factors limiting the transport of microorganisms and viruses, thereby shaping their assemblages (19). Thus, the microbial and viral distribution is closely linked to the oceanic current patterns and, for viruses, is indirectly regulated by environmental factors that modulate the host availability (20). In turn, viruses are also able to modify the microbial community composition, host metabolic activities, and evolutionary trajectories through lateral gene transfer events (21, 22).

Moreover, it has been proposed that viruses in oceans follow a seed-bank model with high local diversity and global distribution (12, 23). In this model, viruses (seeds) are passively transported by ocean currents; viral communities are then structured by local conditions and host availability, as supported by recent global surveys (20, 24). Viruses are the largest reservoir of genetic information in the ocean (6, 25). Global surveys have hinted that the SO contains unusually high amounts of novel genetic diversity; however, this region is systematically under sampled (26).

In marine environments, temperature is one of the primary selective pressures on the microbial community, as it affects critical aspects of cell metabolism, such as osmotic regulation, enzyme activity, and kinetics (27). To cope with low temperatures, bacteria and archaea have developed several physiological adaptations, including the acquisition or evolution of cold shock genes/proteins that can counteract the harmful effects of cold by acting as nucleic acid chaperones (28). Other strategies include the production of cryoprotectant solutes that can reduce the freezing point of the cytoplasm and eliminate free radicals, producing antifreeze proteins that prevent nucleation of ice in the membrane or cytoplasm, and structural modifications to increase membrane fluidity and thicken the peptidoglycan layer (29–32). Other molecular adaptations include changes in component frequencies at nucleotide or protein levels that improve the performance of DNA metabolism or increase the efficiency of enzymatic processes at low temperatures (27). For example, a common mechanism is related to changes in the frequencies of amino acids toward a lower arginine and proline content, which tends to reduce the number of hydrogen bonds and salt bridges (33). Another strategy comprises a general increase of small and neutral chain amino acids in the loop regions of the secondary protein structure, thereby enhancing enzyme flexibility. Among environmental factors, temperature has the strongest correlation with the viral community composition of epipelagic marine viruses (20, 24, 26). However, no strategy for viral adaptation to low temperatures has been reported or characterized to date.

Indeed, the relative isolation and intense selective pressures of the SO, including low temperature and other environmental factors, make Antarctic marine waters a natural laboratory for the study of viral diversification and adaptation. Consequently, we hypothesized that SO viruses present a shared set of adaptations, due to positive environmental selection, and dispersal limitation. To test this hypothesis, we generated a viral genome data set of previously undescribed viruses from the SO to assess the community structure and diversity within a genomic and ecological context. Our results demonstrate that the SO viral communities contain a high number of divergent species from widespread marine viral families. Predicted hosts from SO viruses were the abundant and characteristic members of bacterial phyla associated with phytoplankton blooms that thrive in the region during the summer.

Furthermore, functional analyses of the viral proteome allowed us to identify specific genes associated with physiological and molecular adaptive mechanisms to low temperature. These genes showed enrichment in functions related to the cold-shock-event response and quorum-sensing ability in lysogenic viruses, highlighting an explanatory mechanism for the lytic-lysogenic switch in the SO. Finally, molecular analysis of the Antarctic viral proteome unveils overall changes in the hydrophobicity patterns and specific amino acid frequencies, suggesting temperature-driven genetic selection, as has been seen in cold-adapted cellular organisms.

Together, these findings comprise novel insights to elucidate the novel and adaptive features developed by viral communities to improve their fitness and succeed in the cryosphere.

## RESULTS AND DISCUSSION

### Novel viral populations in Southern Ocean communities.

Major sequencing efforts in recent global marine surveys, such as the Tara Oceans and Malaspina expeditions, have exponentially increased our understanding of the diversity and biogeography of viruses in the ocean environment, particularly concerning the well-sampled oceanic surface waters (23, 25, 34). Nevertheless, some large water masses, such as the advection-limited and isolated SO, remain widely understudied compared to the rest of the oceans, with only a few locations and samples from the SO represented in global viral diversity pattern studies (20, 24).

Here, we collected and analyzed 64 publicly available metagenomic data sets from the SO (60 from cellular and 4 from viral fractions). Additionally, we generated a new metagenomic data set from the viral fraction of Chile Bay (West Antarctic Peninsula) during February 2016, through serial filtration and further CsCl gradient purification, which primarily targets double-stranded DNA (dsDNA) tailed viruses from the *Caudovirales* group (35). Altogether, this allowed us to expand the number of sampling locations and viral sequences from the SO to assess community composition differences while also allowing us to investigate common genetic elements and protein features of the SO viral communities.

A total of 2,548 nonredundant viral draft genomes were recovered from the SO metagenomes and viromes. Their sizes ranged from 10 kb to 279.2 kb, with an average GC content of 43.08%. Known viral species were identified via nucleotide alignment against the Global Ocean Virome data set (GOV2), the most complete available data set of dsDNA marine viruses to date (24). To determine nonredundant viral species, sequences were clustered at >95% average nucleotide identity across 80% of the genome, as previously described (36). Approximately 90% of the retrieved viral genomes from the SO were not present in the GOV2 data set. After removing the redundant sequences, this resulted in 2,416 genomes representing novel viral species, from which 107 genomes were obtained from our newly sequenced SO viromes, while 2,309 were obtained from publicly available data sets not previously mined for viruses. Altogether, this represents approximately a 25% increase in the SO viral genomes with regard to the GOV2 data set.

### Global biogeography of Southern Ocean viruses and diversity.

A combined data set of the sequences recovered in this study and the GOV2 viral genomes was used to calculate the abundance of each viral species through read mapping. Subsequently, a normalized count matrix was used to calculate the diversity metrics and to compare the community structure between viral communities of the SO and other epipelagic samples from the oceanic regions defined in the TARA Ocean virome study (20). The calculated Shannon-Wiener index (H') showed the lowest values in the SO samples (mean H' = 2.54), while the highest values were observed at the equatorial and subtropical regions (mean H' values 4.5 and 4.3, respectively) (Fig. S1 in the supplemental material). The observed pattern is consistent with recent macrodiversity (intercommunity diversity) global patterns observed for viruses, where low diversity values were observed in the Antarctic macroecological zone. In contrast, the highest diversity was observed in temperate and tropical epipelagic waters, followed by the Arctic region (24). This pattern could be strongly influenced by the microbial community composition, and previous studies have shown a negative correlation between species richness and latitude, with a decrease in the microbial community richness from tropical to polar marine environments (37–39).

10.1128/mSystems.00396-21.1 FIG S1 Alpha diversity box plots showing Shannon-Wiener index values (H'). Values were calculated for each individual sample within the different oceanic regions considering the normalized abundances of each virus (for details see the Material and Methods). Download FIG S1, TIF file, 0.2 MB. Copyright © 2021 Alarcón-Schumacher et al. 2021 Alarcón-Schumacher et al. https://creativecommons.org/licenses/by/4.0/ This content is distributed under the terms of the Creative Commons Attribution 4.0 International license.

**FIG 1:**
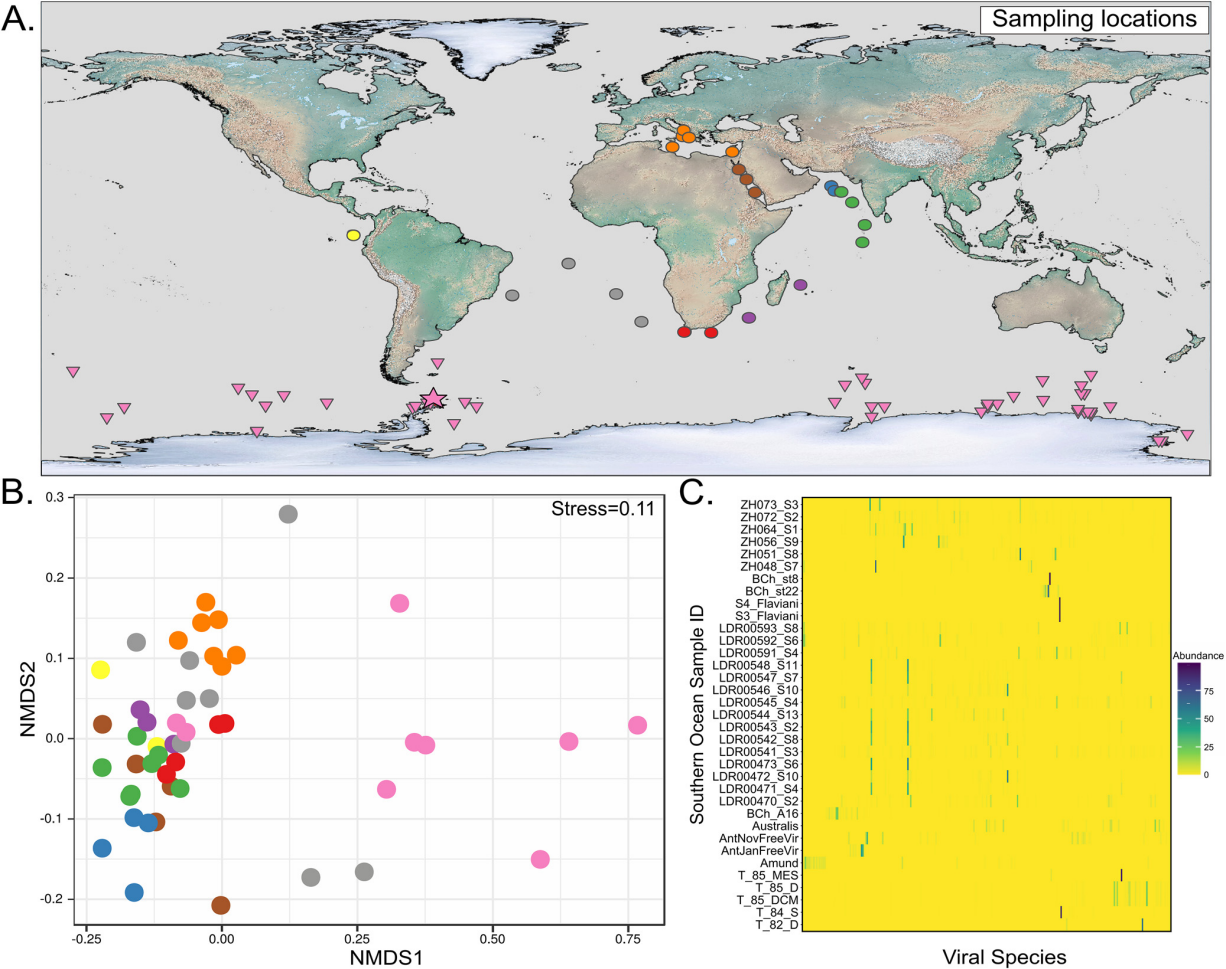
Geographical distribution of Southern Ocean samples. (A) Sampling sites considered in the study. Southern Ocean samples are represented in magenta (samples obtained in this study by magenta star; available metagenomic data from previous studies in the Southern Ocean, magenta triangles). Other oceanic provinces are highlighted in different colors: red, East Africa Coastal (AFC); blue, North West Arabian Upwelling (ARS); green, Indian Monsoon Gyres (IMG); purple, Indian South subtropical gyre (ISG); orange, Mediterranean Sea (MED); yellow, N. Pacific Equatorial Countercurrent (PEC); brown, Red Sea (RS); gray, South Atlantic Gyre (SATL). (B) Nonmetric multidimensional scaling (NMDS) analysis was constructed based on the Bray-Curtis distance matrix of viral genomes. The Bray-Curtis distances were calculated from normalized viral abundances. (C) Relative abundances of the viral community from Southern Ocean samples (only viral species with relative abundance of >1% across all samples are displayed).

Compositional dissimilarities calculated from the abundance matrix were used to compare viral communities. Nonmetric multidimensional scaling (NMDS) (Fig. 1B), as well as hierarchical clustering (Fig. S2), revealed a general grouping of epipelagic SO viral communities distinct from the rest of the viral communities of other oceanic provinces. All SO viruses formed a compact cluster except for the sample T_MES_85, which is highly divergent and corresponds to the sole available mesopelagic viral data set from the SO. This suggests that surface and mesopelagic viral communities may change drastically, despite the strong upwelling processes taking place in the SO, and further research of these communities could reveal an even larger unknown viral diversity.

10.1128/mSystems.00396-21.2 FIG S2 Hierarchical clustering analyses of the viral operational taxonomic unit (OTU) abundance matrix with samples from the Southern Ocean (highlighted in purple) and the samples from the TARA ocean series from different oceanic provinces. Download FIG S2, TIF file, 0.4 MB. Copyright © 2021 Alarcón-Schumacher et al. 2021 Alarcón-Schumacher et al. https://creativecommons.org/licenses/by/4.0/ This content is distributed under the terms of the Creative Commons Attribution 4.0 International license.

An additional k-mer frequency analysis of exclusively unassembled virome samples was performed to avoid putative compositional biases. Principal-component analysis (PCA) using Bray-Curtis dissimilarity distances calculated from tetranucleotide frequencies (as a proxy for genetic differentiation) exhibited a very similar ordination, with divergent clustering of the SO viral communities in comparison to the other viral communities from the oceanic regions studied in this work. Together, these differences in community composition and genetic distances based on k-mer frequencies suggest that the SO harbors unique endemic viral communities, as was previously suggested by other Antarctic studies and global surveys (12, 20). We hypothesize that these differences might arise from the relative physical isolation of the SO due to the Circumpolar Antarctic Current, as has already been observed for microbial communities of a transect crossing the Antarctic Polar Front (18). However, further studies in the SO are needed to confirm this potential viral endemism.

Interestingly, ordination and clustering analyses showed that the viral community structure presents a high compositional variation between SO locations (Fig. 1B and Fig. S2). The calculated abundances for SO viruses in our data set show that only a few species were highly abundant in each sample, with relative abundances lower than 1% for most of the recovered viral genomes (Fig. 1C). Conversely, low values of evenness were observed across the different samples in the SO. Furthermore, in Chile Bay (West Antarctic Peninsula) during a period of phytoplankton bloom, particularly low values were observed (Pielou’s 0.04 and 0.27, for samples BCh_st8 and BCh_st22, respectively). This period was characterized by a microbial community dominated by only a few bacterial clades, the *Pseudomonadales* and *Alteromonadales*, which resulted in a highly uneven viral community with a small number of viral genomes present in the samples, as reported in reference 8. In contrast, several viral species were found to be abundant in a sample taken during November from the West Antarctic Peninsula (Pielou’s evenness = 0.82, Table S2), where no phytoplankton bloom was observed and therefore no bacterial clade was expected to be reproducing at rates that would allowed a single viral species to become increasingly dominant in the community. Notably, most of the metagenomic studies of the SO have been carried out during the austral spring and summer seasons, which are periods characterized by large phytoplankton blooms often dominated by single phytoplankton and bacterioplankton species (5). This combined with the strong seasonality could bias the observed viral community structure such that further research is thus needed to better understand the yearly changes in the viral communities of the SO.

10.1128/mSystems.00396-21.5 TABLE S2 Relative evenness (Pielou’s J) of the detected viral populations at each sample site in the Southern Ocean. Download Table S2, XLSX file, 0.01 MB. Copyright © 2021 Alarcón-Schumacher et al. 2021 Alarcón-Schumacher et al. https://creativecommons.org/licenses/by/4.0/ This content is distributed under the terms of the Creative Commons Attribution 4.0 International license.

### Genome-based viral taxonomy estimations.

**FIG 2:**
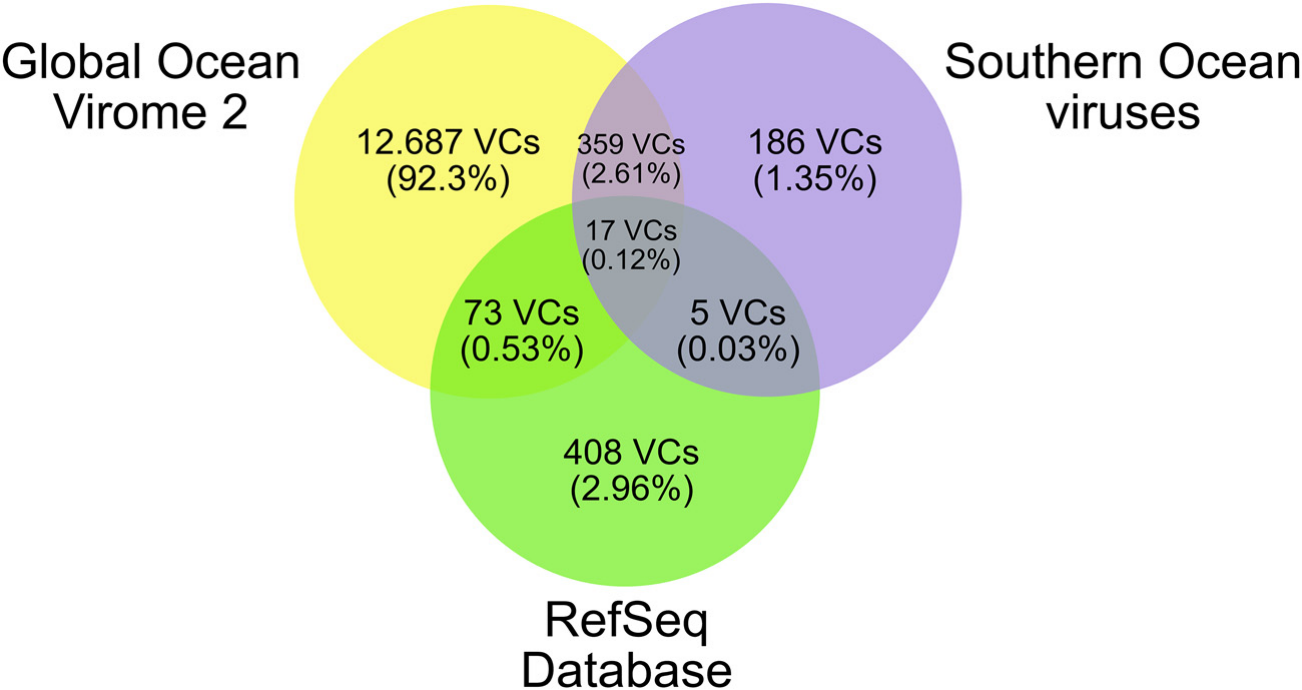
Clustering of viral populations. Venn diagram representing the three different source data set for the viral genomes. Viruses were classified into viral clusters (VCs) using vContact2. The number of clusters is displayed in the circles and the percentage in parentheses represents the proportion of the total clusters. Only VCs with at least two different viruses were considered.

Even though the current prevailing notion is that the epipelagic marine viral diversity is relatively well sampled (23, 25, 34), our results suggest that many viral species seem to be unique to the SO. We hypothesized that most of the newly recovered viral genomes from the SO do not actually constitute new viral genera but instead represent highly divergent members of known viral clades that have been under strong selection due to geographic isolation and the environmental pressures of Antarctica. To demonstrate this, and in the absence of a proper/universal viral phylogenetic marker (40), a taxonomic classification was performed using a shared protein-based algorithm (41). First, we clustered all predicted viral proteins from the genomes of the SO with those from the complete viral RefSeq proteins (release 94) using vConTACT2(42, 43). These clusters were used to group the genomes based on shared protein clusters, yielding viral clusters (VCs), which are suggested to be approximately equivalent to a genus- or family-level taxonomic classification of the International Committee on Taxonomy of Viruses (ICTV) (25, 42, 43). The SO viruses were clustered with the GOV2 viral genomes (25), which resulted in the majority of SO viral genomes forming VCs with other marine viral sequences from the GOV2 data set (Fig. 2). Interestingly, 186 VCs (genera) were unique to the SO, representing 421 unique viral genomes (17.4% of the novel reported viral species). These results support the hypothesis that most of the viral diversity observed in the SO viral communities corresponds to divergent groups of widespread marine viruses. The assessment of environmental drivers of community structure demonstrates that the temperature variable exhibited the highest positive correlation (Mantel *r* statistic = 0.56, *P* < 0.001) followed by the oceanic region (Mantel *r* statistic = 0.23, *P* < 0.001). This pattern also agrees with that observed at the SO intraregional level, where temperature appears as the primary determinant of variability among viral communities (44). Subsequently, we hypothesize that present-day SO viruses arose via the strong environmental selective pressures of the SO region and the low gene flow associated with the circumpolar current. Since the effects of temperature and geographic distance are well-known determinants of microbial communities (20, 26), we further investigated the physiological and molecular adaptations of SO viral communities under these environmental and physical constraints.

### South Ocean virus-prokaryotic host associations.

To determine the potential hosts within the prokaryotic communities for the novel viruses obtained, we performed *in silico* host prediction using VirHostMatcher-Net, a network-based approach that integrates CRISPR sequences and alignment-free similarity measures. More than 4,000 bacterial and archaeal nonredundant genomes from marine environments deposited in the NCBI database were investigated as putative hosts (45). VirHostMatcher-Net analysis resulted in 1,106 virus-host pairs predicted (∼45.8% of the total viral community was match) with high confidence levels (minimum overall score 0.95 and no taxonomic ambiguity).

The most common hosts found represent abundant and relevant bacterial clades in the SO, headlined by the *Flavobacteriia* with 682 virus-host pairs (e.e., *Aquimarina* and *Flavobacterium*), followed by *Alphaproteobacteria* with 179 pairs (e.g., *Sphingomonas*), *Gammaproteobacteria* (e.g., *Pseudoalteromonas*), and *Actinobacteria* (e.g., *Streptomyces*) with 111 and 43 pairs, respectively. These groups, and in particular the *Flavobacteriia*, are known to be strongly linked to the phytoplankton blooms that take place during the summer season in the SO, increasing their abundance rapidly in response to the higher availability of organic matter and the increase in temperature (5, 46, 47). However, the accuracy of virus-host pair prediction methods remains highly controversial and thus further interpretation at lower taxonomic levels must be carefully considered, especially in environmental systems that are generally underrepresented in databases and culture collections.

### Southern Ocean viral physiological adaptations.

Viruses are capable of reshaping the metabolism of their host during infection cycles. This can profoundly impact the translation of key host proteins, affecting energy pathways, substrate uptake, and ion exchange. Downregulation of these pathways can be detrimental to viral fitness. Therefore, viruses usually carry genes that can replace the host proteins of critical metabolic processes to ensure the minimum conditions for host survival are met during viral replication, as has been observed for photosynthesis pathways in cyanobacterial viruses (48, 49).

When comparing the overall profile of the SO viral proteins with the global oceanic profile (Fig. 2), a high similarity was found. Proteins related to replication and repair of DNA were the most abundant in both data sets (30 and 33% of the assigned proteins for GOV2 and SO, respectively), followed by cell wall and membrane biogenesis (20 and 16%, respectively). An assessment of auxiliary metabolic genes (AMGs) with VIBRANT (50) revealed that genes related to amino acids, cofactors, and vitamin metabolism were the most abundant AMGs (e.g., genes involved in cysteine and methionine metabolism [71 AMGs] and folate biosynthesis [21 AMGs]). On the other hand, most of the predicted viral proteome in the SO has an unknown function. The functions detected with the VIBRANT software in the SO viruses have already been commonly observed in marine viruses, and therefore their presence alone does not necessarily constitute evidence of a particular adaptive trait in SO.

To identify viral genes under positive selection in the SO, we selected protein clusters with a minimum size of 50 proteins and containing both GOV2 and SO proteins. Then, the frequency of clusters within the SO viral data set was calculated and compared to the global ocean data set clusters. A total of 30 SO clusters had a frequency at least 5 times higher than the ocean average (mean = 6.93, standard deviation [SD] = 1.47).

The functional profile revealed a high frequency of domains involved in DNA metabolism, such as DNA-binding proteins, DnaB helicases, and DNA methylases (Pfam domains PF12684, PF03796, and PF01555, respectively). Several proteins related to nucleic acid metabolism, as well as the DNA chaperones and chaperonins that mediate nucleic acid and protein folding, are preferentially expressed in response to major cold shock events in bacteria (51, 52). This suggests that SO viruses carry genes analogous to those present in their host to ensure host survival during viral replication. Additionally, most of the SO viruses encode at least one analog of the GroEL/GroES complex (Table S3). These chaperonins are also ubiquitous in bacteria and archaea and actively participate in protein folding, particularly under nonspontaneous folding conditions. Interestingly, the GroEL/GroES complex has been experimentally demonstrated to confer the ability to grow at low temperatures in mesophilic organisms (53). Although this protein complex has been previously reported in other marine viruses, its high frequency in the SO viruses suggests that this trait could be subjected to positive selection in this particular environment.

10.1128/mSystems.00396-21.6 TABLE S3 Virally encoded auxiliary metabolic genes and other genes of interest related to cold-shock response. The cold response reported activity to which each enzyme is related and the viral homologs in the Southern Ocean dataset are listed. Download Table S3, XLSX file, 0.01 MB. Copyright © 2021 Alarcón-Schumacher et al. 2021 Alarcón-Schumacher et al. https://creativecommons.org/licenses/by/4.0/ This content is distributed under the terms of the Creative Commons Attribution 4.0 International license.

**FIG 3:**
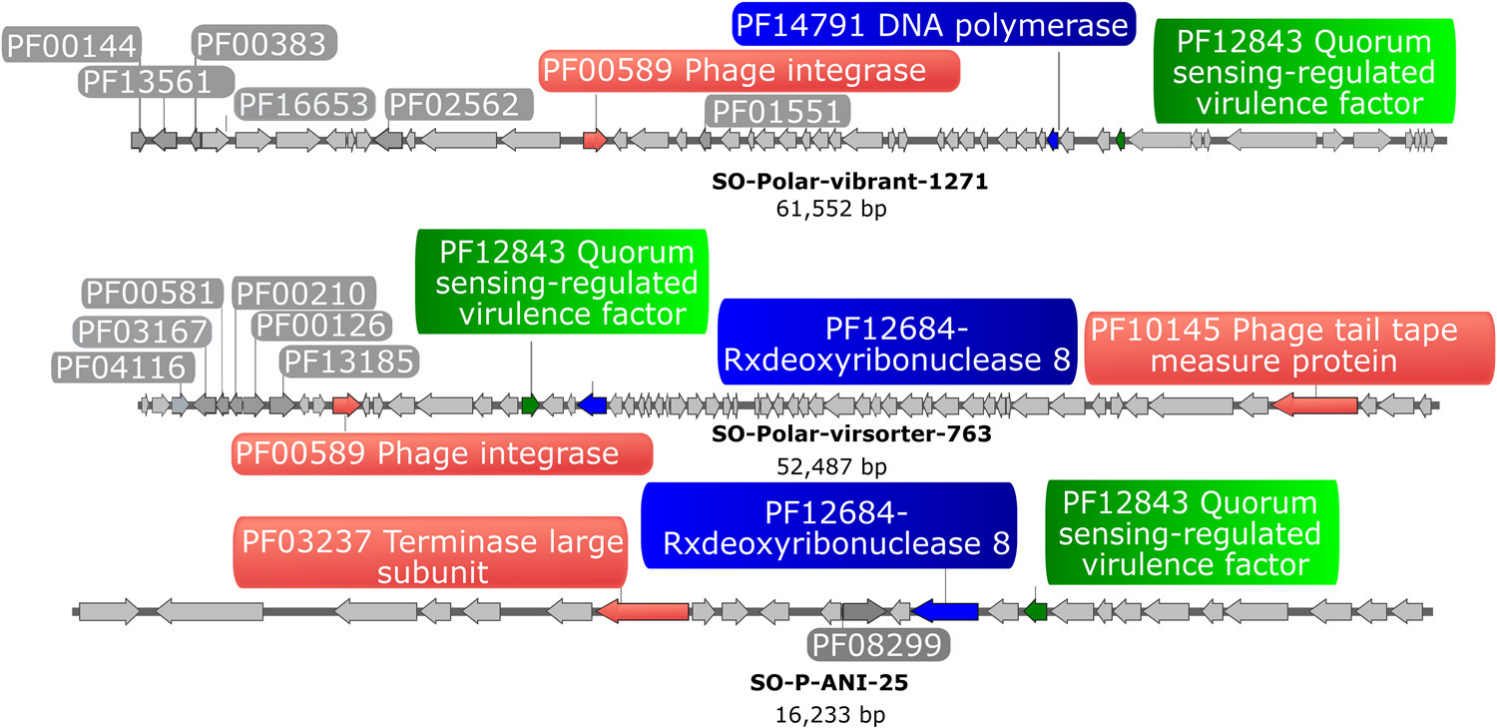
Positively selected and frequent Southern Ocean genes. The figure shows three retrieved viral genomes to exemplify prevalent encoded genes among SO viruses. Green and blue colors represent frequent Pfam motifs in SO viruses, while red-highlighted genes are predicted structural or hallmark viral genes. Genes highlighted in gray are additional Pfam annotations within these viral contigs.

Another protein cluster that appeared to be under positive selection was a quorum-sensing regulated group of proteins (domain PF12843), which was previously identified in Pseudomonas as a putative quorum-sensing-regulated virulence factor (54). Detailed analysis of the genomic context showed that genomes that encoded the quorum-sensing-related gene often also encoded integrase genes (Fig. 3). Quorum-sensing response mechanisms are widely distributed among viruses, with some phages presenting a communication system denominated by arbitrium, which is similar to quorum sensing but involves small signaling peptides (55). However, this system also presents inherent stochastic factors and the mechanisms driving the lytic-lysogenic decision remain mostly uncovered for the majority of the virosphere. Nevertheless, we hypothesize that a high frequency of these proteins related to quorum sensing enables SO viruses to sense and respond to the strong seasonal changes in temperature and nutritional conditions by modulating the shifts in the lysogenic-lytic cycle decision. However, further experimental data are now required to elucidate the specific role of this protein cluster.

Given that temperature is an intense selective pressure in the SO, we hypothesized that genes related to common prokaryotic adaptive strategies to the cold environments would be frequent among the SO viruses. Unexpectedly, only a few analogous to some of the most common and well-characterized bacterial cold shock proteins related to the Csp family (Table S3), which act as nucleic acid chaperones (30), were detected in the SO viral genomes. Similarly, a near absence was observed for other common biosynthetic pathways involved in bacterial cold adaptation, such as trehalose production or analogs of desaturases genes, which can increase membrane fluidity by introducing double bonds into fatty acids. Simultaneously, only one antifreeze protein, which prevents ice nucleation in the cell, was detected (Table S3). Notably, we detected several analogs of the gene encoding uridine diphosphoglucose pyrophosphorylase (UDPGP), which participates in cell wall polymer and exopolysaccharide (EPS) biosynthesis, modulates cell permeability, and lowers ice nucleation temperature. The UDPGP gene could be part of a cryoprotectant mechanism encoded by the viruses to improve host survival during infection and ultimately increasing viral fitness.

### Molecular adaptations of viral Southern Ocean proteins.

**FIG 4:**
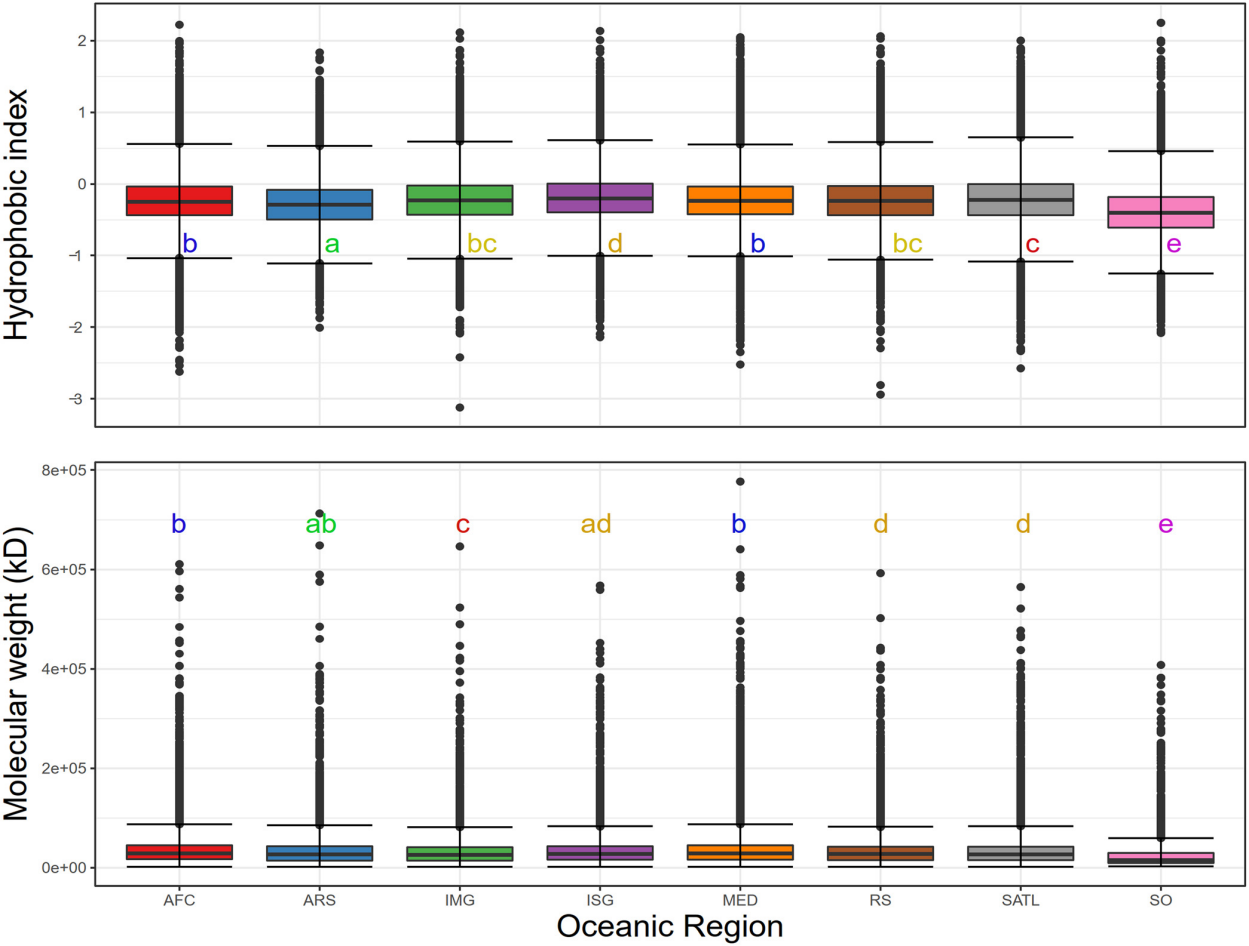
Overall molecular properties of viral proteins. Statistical analyses (Kruskal-Wallis and Wilcoxon paired tests) of physicochemical protein properties (hydrophobicity index and molecular weight) of predicted viral proteins. Letters represent different groups defined by the Wilcoxon paired test.

Psychrophilic enzymes in the SO must cope with average temperatures of approximately −1 to 1°C in the winter and 3 to 5°C in the summer (56, 57). This critical factor reduces biochemical reaction rates; therefore, enzymes must develop suitable adaptations to remain efficient enough to maintain cellular metabolism (33, 58). Known adaptations to low temperatures include changes in nucleotide utilization, codon usage, amino acid content, protein structure, and enzymatic affinity. Because of the different nature of these adaptations, and to avoid putative bias of observing a site-specific mutation or single virus variation, we analyzed the global protein physicochemical properties (i.e., molecular weight, hydrophobicity index, and isoelectric point). This overall approach allowed us to summarize in single indexes transversal adaptations across the different viral genomes. Statistical analyses (Kruskal-Wallis and Wilcoxon paired tests) revealed significant differences (*P* < 0.01) between SO and other ocean province viral proteins (Fig. 4), and additional size effect by the Cohen’s *d* comparison (Table S4). SO viral protein properties showed lower hydrophobic index values (mean = −0.39; SD = 0.41) (Table S4) compared to other regions, which were up to two times higher. Size effect calculations showed that the magnitude of these differences was small to medium (average Cohen’s *d* = 0.45), with a 62.5% chance that, when picking a random viral protein from the SO, the hydrophobicity index will be lower than a protein selected from any other ocean province. This lower hydrophobicity index pattern is typically observed for cold-adapted bacterial proteins, where a reduced amount of buried hydrophobic residues affect folding processes and increase protein flexibility, a key aspect to effective performance at lower temperatures (59, 60). Although the hydrophobic index measurement performed here is based on whole-protein sequences, rather than explicitly targeting buried amino acids, it suggests smaller hydrophobic cores and less rigid structures. In fact, as the magnitude of the observed differences was not trivial, this provides new evidence to suggest that these viral protein adaptations occurred to gain structural flexibility and efficiency in the cold waters of the SO.

10.1128/mSystems.00396-21.7 TABLE S4 Summary of physicochemical properties (hydrophobicity, isoelectric point, molecular weight, and half-life) and amino acid frequencies of the viral proteome of each oceanic region. Size effect values (Cohen’s *d*) of the comparisons between the SO proteome and each oceanic region are listed. Download Table S4, XLSX file, 0.02 MB. Copyright © 2021 Alarcón-Schumacher et al. 2021 Alarcón-Schumacher et al. https://creativecommons.org/licenses/by/4.0/ This content is distributed under the terms of the Creative Commons Attribution 4.0 International license.

Significant differences were also observed for molecular weight (MW), with lower values and a narrower range observed in the SO (mean = 24,743.87 Da; SD = 28,111.49, range= 2,347.63 to 407,958.64 Da) (Table S4). However, size effect calculations estimated that the magnitude of the difference was small in all cases (average Cohen’s *d* = 0.32), with a 59% probability of obtaining a lower MW value for a random viral protein from SO. Previous studies have shown a direct correlation between a high MW and the number of cavities in globular proteins, which translates to a higher energetically unfavorable packing probability (61). While a poor correlation has been observed for psychrophilic enzymes (62), we propose that the lower MW observed for SO viral proteins could decrease erroneous packaging frequency in an environment where high salt concentrations and low temperatures have a direct impact on protein folding and kinetics (33). Several threads of evidence presented in this work suggest that genes related to protein folding (i.e., chaperonins), as well as the variants of core proteins with reduced hydrophobic cores and lower MW, are under positive selection in the SO virome.

Since studies addressing different structural and functional adaptation strategies of viral proteins to cold are scarce, we also compared the observed patterns of amino acid frequencies in SO viral proteins against other oceanic regions’ proteomes. Many of the known adaptation mechanisms in psychrophilic bacteria try to increase the structural flexibility of their proteins and thereby enhance the degree of complementarity between the substrate and the active site, reducing the required activation energy and improving turnover rates (33, 63). This increased flexibility and catalytic activity can be achieved by modifying the amino acid frequencies of specific residues in the proteins. This strategy aims to reduce the number of salt bridges and hydrogen bonds between the secondary structures, minimize the interaction between stacking aromatic rings, and generally use smaller or neutral residues in the loop regions of the secondary structures, among others (27, 64).

**FIG 5:**
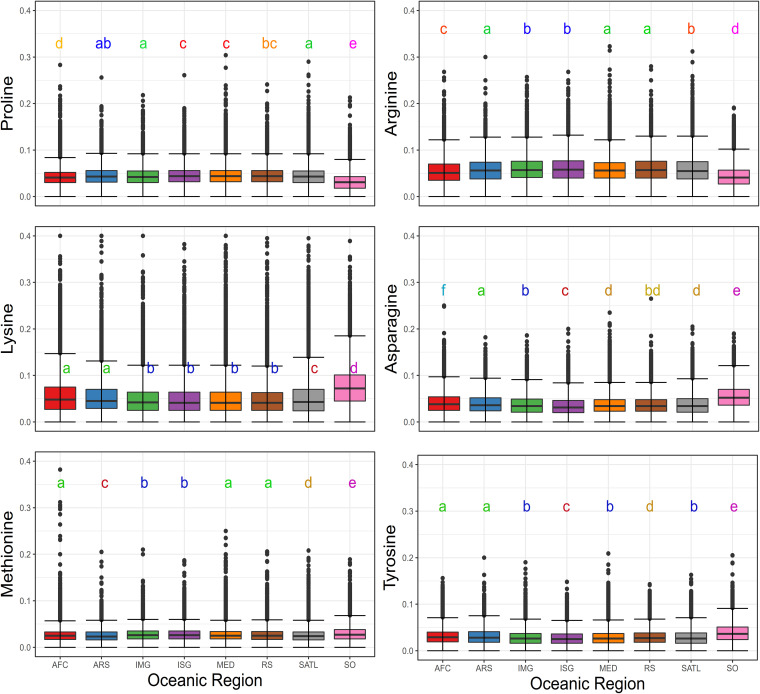
Relative amino acid frequencies of viral proteins. Kruskal-Wallis test and subsequent pairwise comparisons using the Wilcoxon rank sum test on amino acid frequencies in viral proteins from different oceanic regions. Amino acids with significant statistical differences and biological implications for cold adaptation are displayed (arginine, proline, asparagine, lysine, methionine, and tyrosine). Letters represent different groups defined by the Wilcoxon paired test.

Significant differences (Kruskal-Wallis and Wilcoxon paired tests) in the frequencies of several amino acids were observed in the SO virome, compared with their mesophilic homologues. For instance, proline showed a lower frequency (mean = 0.03; SD = 0.02) in SO viral proteins (Fig. 5 and Table S4), with a medium magnitude of change (average Cohen’s *d* = 0.57). Lower frequency of proline is a common trait observed in cold-adapted proteins of psychrophilic bacteria (i.e., beta-lactamases and aminopeptidases) (33). During protein folding, prolyl residues can adopt only a few conformational configurations that contribute to the local rigidity of the protein backbone (65, 66). Therefore, the reduced proline content enhances the chain flexibility of protein secondary structures and reduces the rate-limiting step of proline isomerization during protein folding (67). In psychrophilic bacteria, this step is usually achieved by prolyl isomerase trigger factor activity (68); however, there were no detectable analogs for this enzyme among the SO viral proteins (Table S3). This suggests that viral genomes could have reduced their proline content as a more cost-effective strategy than encoding the enzymatic machinery required to improve protein folding.

Arginine was also present at lower frequencies in the SO viral proteins (mean = 0.04; SD = 0.02), while lysine presented higher values (mean = 0.08; SD = 0.05) with a magnitude of the difference considered from medium to large (average Cohen’s *d* = 0.54 and −0.68, respectively). Commonly, the presence of arginine in proteins is considered a stabilizing agent because it increases the number of salt bridges and consequently promotes structural stability and rigidity. It has been shown that in cold-adapted proteins, arginine is often replaced by lysine, resulting in a higher lysine-to-arginine ratio. The subsequently decreased hydrogen bonding and salt bridge formation enhance protein flexibility and activity at low temperatures (68, 69). Our analysis shows that SO viral proteins present significant differences in amino acid composition (Fig. 5) and a higher lysine-to-arginine ratio (Fig. S4) compared to viruses from other regions. Therefore, this strategy seems to be a widespread adaptation among SO viral proteins.

Additionally, higher frequencies of asparagine (mean = 0.05; SD = 0.03; medium to large magnitude of change; average Cohen’s *d* = 0.7) and methionine (mean = 0.03; SD = 0.02; small magnitude of change; average Cohen’s *d* = −0.2) were also observed in SO viral proteins. These are common compositional biases observed in cold-adapted proteins (65, 68, 70), even though their effects have not been extensively studied. The observed differential content might be due to a lower or absent constraint on temperature-driven deamination, and therefore no negative selection against asparagine frequency occurred. In thermophilic proteins, thermo-inactivation in α-amylase caused by deamination of asparagine or glutamine, or both, residues has been shown experimentally. The decrease of these amino acids in thermophilic proteins has been shown to avoid detrimental effects of temperature (71, 72). As for methionine, it has been proposed that in some cold-adapted enzymes, such as trypsin, the conservation of four methionine residues provides additional flexibility to the cleavage active site (73). However, the magnitude of change is small in our data set, suggesting that the change in frequency affects only particular families of proteins and is not a widespread adaptation.

Interestingly, tyrosine residues also exhibited an increased frequency (mean = 0.04; SD = 0.02) compared to the ocean averages (medium magnitude of change; average Cohen’s *d* = −0.5). Tyrosine increase could have an essential role in protein folding, as it influences the conformation and rigidity of multilayer stacking in β-barrels and can be extremely conserved in certain positions, such as the “Tyr corner” in Greek key proteins (74, 75). However, we suggest that the hydrophobic characteristic of tyrosine is not subjected to negative selection pressure, given that at temperatures below 25°C, the hydrophobic effect of aromatic and hydrophobic residues weakens (76). The latter point is also supported by the frequency of other aromatic amino acids, where no negative selection was observed for tryptophan or phenylalanine compared with the ocean averages (Fig. S3).

10.1128/mSystems.00396-21.3 FIG S3 Extended amino acid relative composition of viral proteins and their statistical differences. Download FIG S3, TIF file, 1.1 MB. Copyright © 2021 Alarcón-Schumacher et al. 2021 Alarcón-Schumacher et al. https://creativecommons.org/licenses/by/4.0/ This content is distributed under the terms of the Creative Commons Attribution 4.0 International license.

In conclusion, the Southern Ocean is an environment characterized by its physical isolation and extreme selective pressures, such as low temperatures and strong seasonality, compared to the rest of the global oceans. In order to survive these conditions, local populations have developed multiple adaptive mechanisms. In the present study, we show that the SO harbors a unique diversity of viruses that have developed specific adaptations to cope with low temperatures. These viral adaptations are mainly associated with protein folding and increased protein flexibility to enhance enzymatic activity under less thermodynamically favorable conditions. Viruses ensure their host survival during the infective cycle by encoding several genetic elements, such as chaperones and nucleases. They also commonly present quorum-sensing-related genes, which may play key roles in the lytic-lysogenic shift decision. Furthermore, changes in specific amino acid frequencies likely allow viruses to increase their proteins’ flexibility and enhance their enzymatic activity under cold environmental conditions. Altogether, our results suggest that this skew in the coding frequencies of specific amino acid residues is the most prevalent and transversal strategy of cold adaptation in the SO viral communities. Isolating new psychrophilic viruses and studying their adaptations are essential to test the proposed hypothesis. Finally, the discovery of novel cold adaptation mechanisms in viruses will broaden our understanding of how viruses remain successful in the extreme conditions of polar marine environments.

## MATERIALS AND METHODS

### Sample collection and processing.

Sampling was conducted on a weekly basis in February 2016 at Chile Bay (62° 27'6” S; 59° 40'6” W), Greenwich Island, South Shetlands Islands, West Antarctic Peninsula (Fig. 1A). Five samples (altogether ∼100 liters) of seawater from a 2-m depth were collected weekly throughout the month. The water was first filtered through a 200-μm polyester net and then transported in darkness to the laboratory for serial filtration using a Cole Palmer System peristaltic pump (model no. 7553-70; 6 to 600 rpm; pressure up to 200 kPa). Distinct cellular fractions were separated using 20-μm filters, 8-μm polycarbonate filters, and 0.22-μm PES Sterivex filters (Millipore, Darmstadt, Germany). Filtered water (<0.22 μm fraction) was ultraconcentrated by tangential flow filtering with a 30-kD membrane Vivaflow (Sartorious) to a final volume of 50 ml, as described in Guajardo-Leiva et al. (77). Viral concentrate was purified by CsCl gradient as previously described (78). Briefly, gradients were prepared in thin wall polypropylene tubes (Beckman Coulter) by adding 1 ml of the CsCl solutions (densities 1.7, 1.5, 1.35, and 1.2 g/ml) and loading 3 ml of sample into each tube, adjusting the density to 1.12 g/ml. The tubes were then ultracentrifuged at 21,755 rpm for 2 h using an SW 40 Ti swinging-bucket rotor (Beckman Coulter). Viruses were resuspended in sterile seawater and treated with a TURBO DNA-free kit (Invitrogen) (20 U/ml, 1 h at 37°C). Subsequently, DNA was extracted using a formamide/CTAB protocol (79). The integrity and purity of the DNA was assessed through 1% agarose gel electrophoresis and PCR targeting the V4 hypervariable region of the 16S rRNA gene using the primer pair PE_16S_V4_515F and PE_16S_V4_786R, according to a described method (80). Purified viral DNA from the weekly samples was then pooled for sequencing.

### Nucleic acid sequencing and bioinformatics.

DNA was sequenced at the Roy J. Carver Biotechnology Center, W.M. Keck Center for Comparative and Functional Genomics (University of Illinois). Paired-end libraries were generated using the KAPA DNA sample preparation kit and sequenced on one MiSeq flowcell for 251 cycles from each end of the fragments, using a MiSeq 500-cycle sequencing kit version 3. Fastq files were generated and demultiplexed with the bcl2fastq v2.17.1.14 Conversion Software (Illumina). PhiX genomic (3 kb) DNA was used as a spike-in control for the MiSeq run.

### Southern Ocean viral contig identification.

Sequenced samples from this study and publicly available SO cellular and viral metagenomic data from previous studies (Fig. 1A) were used to identify viral sequences and create an enriched database with SO viruses, which is summarized in Table S1.

10.1128/mSystems.00396-21.4 TABLE S1 Environmental metadata for marine metagenomic datasets. Reference study and sequencing technology are also listed. Download Table S1, XLSX file, 0.01 MB. Copyright © 2021 Alarcón-Schumacher et al. 2021 Alarcón-Schumacher et al. https://creativecommons.org/licenses/by/4.0/ This content is distributed under the terms of the Creative Commons Attribution 4.0 International license.

For cellular and viral metagenomic samples obtained from previous studies, the following procedure was applied: quality control of reads (Q > 30) was done using Cutadapt software (81), while reads matching the PhiX Control were *in silico* removed prior to assembly. Samples were then assembled using MEGAHIT (v1.0.6) (82) with the “meta” preset (all other parameters were set as default). Because of the low number of reads per sample, as well as coverage and depth limitations due to the sequencing technology (titanium 454), an additional coassembly step was performed for the 35 sites from the Aurora Australis Survey. The coassembly was performed using MEGAHIT with the same presets by pooling together all reads to identify additional viral contigs. Contigs over 10 kb, as well as circular contigs, were analyzed using three viral prediction tools that use different algorithms to enhance detection. Specifically, VirSorter software uses reference-dependent and reference-independent approaches, leveraging probabilistic models (83); MARVEL software uses a random forest machine learning approach (84); and the VIBRANT tool uses neural network machine learning of protein annotation signatures (50). Predicted viruses from categories with no significant hits against bacterial or archaeal genomes were considered (BLASTn E value of <1e−10 and coverage of <5%). Prophages from VirSorter categories four to six were manually curated in order to exclude contamination from bacterial genomes. Selected prophage contigs were rechecked with the phage search tool PHASTER (85) to further avoid contamination bias and confirm the presence of viral hallmarks. Additional quality and completeness assessment was performed with the software Checkv (86).

### Southern Ocean viral community composition.

Viral contigs were clustered at the species level with NUCmer (87) at ≥95% ANI across ≥80% of their length (20, 36, 88, 89) to generate a set of nonredundant viral genomes. Quality-trimmed reads from each virome and metagenome were mapped with BBmap (90) against the nonredundant viral database, with the parameters ambiguous=random, K = 13, and a minimum identity of 0.95. A viral species was considered present in a sample only when reads from a sample mapped with at least a coverage of 1× across 75% of their genomes, otherwise the abundance was treated as zero (absent). Total read counts were normalized by contig length and by library. Then we used the MetagenomeSeq R package (91) for normalizing the reads, as recommended in reference 36. The resulting abundance matrix was used to calculate Bray-Curtis distances between samples and perform hierarchical cluster analyses. Additional clustering was performed for SO samples with only viruses of relative abundance of >1% within each sample to study the most abundant members of the communities. Diversity index calculations, as well as nonmetric multidimensional scaling (NMDS), Mantel tests, and ANOSIM analyses, were performed with the R Vegan package (92).

To avoid putative bias on the observed results because only contigs/genomes larger than 10 kb were considered, an additional k-mer frequency was computed with the total reads obtained exclusively from purified viral fraction-derived samples. Bray-Curtis dissimilarity and principal coordinates analysis (PCA) were calculated using the software Simka (93).

### Taxonomic classification and viral protein cluster determination.

Proteins were predicted from candidate viral genomes using Prodigal (94), with the metagenomic mode (-p meta), bypassing Shine-Dalgarno sequence (-n) and other default parameters. Functional annotation was performed with Interproscan 5 (95) based on the Pfam database and manually curated. SO viral contigs with a length ≥10 kb and ≥10 predicted proteins were selected for further analyses. The taxonomic classification was performed using two different methods. First, nucleotide alignment against the RefSeq database (v86, 11-03-2018 http://www.ncbi.nlm.nih.gov/refseq/), was performed via BLAST (96), with a minimum hit score of 50 and an E value of 10^−10^. Second, a gene-sharing networks algorithm was used to annotate the viral taxonomy implemented in vContact2 software (42, 43) using the Viral RefSeq-prokaryotes-v85.ICTV database as a reference and other default parameters.

Due to the low number of classified viruses, we further expanded the analysis by adding all available genomes and proteins from the RefSeq database, as well as the candidate viral contigs from the genomic viral data set “Global Ocean Virome 2” (GOV2) (24). This data set includes thousands of uncultured viral genomes obtained from the assembly of samples from the Tara Oceans series and the Malaspina expedition that were deposited into the database. All-vs-all BLASTp was performed with DIAMOND (97) using a minimum bit score of 50 and an E value of 10^−5^. Protein clusters were defined using the Markov clustering algorithm MCL (41) implemented in vContact2 (42) as previously described (25). The resulting clusters are proposed to be approximately genus-level taxonomy or, much less frequently, subfamily-level taxonomy (25).

In order to assess the ecological importance of the generated viral protein clusters, an additional calculation of the frequency of each individual cluster in the SO and the rest of the oceanic regions (hereafter referred to as the ocean average) was performed as follows. First, only protein clusters present in ≥50 viruses within the total data set were included, thereby excluding rare protein clusters and singletons. Second, the frequency was calculated as the proportion of viruses carrying a protein from a particular cluster with respect to the total viruses. This analysis was performed separately for the SO viruses and the ocean average viruses. Then, we calculated the ratio between the SO frequency and the ocean average. Protein clusters where the frequencies in the SO were ≥5× the ocean average were considered overrepresented in SO viruses.

### Phage-host prediction.

*In silico* host prediction was performed on the nonredundant set of viral contigs identified previously as SO viruses using VirHostMatcher-Net, which combines network-based, Markov random field framework for predicting virus-prokaryote interactions and combining multiple integrated features, including CRISPR sequences and alignment-free similarity measures (45). The prediction was performed against 4,034 bacterial and archaeal genomes obtained from the NCBI using the options -l “marine list” and the -short-contig, with a number of predictions of 3 (*n* = 3) as recommended by the software authors. Predictions were considered reliable for hits with overall scores having thresholds ≥0.95, which estimates an accuracy of 0.9259 at the class level. The best three hits were considered for manual curation using LCA. Viral contigs with ambiguous assignation at the class level were discarded from the analyses.

### Analysis of protein properties.

The physicochemical properties, including molecular weight (MW), hydrophobicity (Hy), isoelectric point (pI), and amino acid composition, of predicted proteins from viral genomes (∼180,000 proteins) were *in silico* inferred for each sample using the ProPAS tool (98). Samples were grouped according to the Longhurst provinces (99), except for samples from the “Antarctic” (ANTA) and “Austral polar” (APLR) regions, which were unified for comparison analysis purposes under the category “Southern Ocean” (SO) due to their origin. Nonparametric variance analyses were performed using the Kruskal-Wallis test and subsequent pairwise comparisons using Wilcoxon rank-sum test were performed with the R package “multcompView”(https://cran.r-project.org/web/packages/multcompView/) and then plotted with ggplot2 (https://cran.r-project.org/web/packages/ggplot2). Further effect size tests were performed using the Cohen’s *d* test implemented in the r package “effsize” (https://cran.r-project.org/web/packages/effsize/).

### Data availability.

Viral contigs reported in this study, as well as extended additional data analysis files, are available online (Figshare repository: https://figshare.com/projects/Ecogenomics_and_adaptation_strategies_of_Southern_Ocean_viral_communities/80762). Publicly available data sets used in this study are archived in their respective ENA and SRA repositories (see Table S1 in the supplemental material for accession numbers).
