# Supplementary figures and images for: Ecogenomics and Adaptation Strategies of Southern Ocean Viral Communities
Source: mSystems. 2021 Aug 10;6(4):e00396-21. doi: 10.1128/mSystems.00396-21 (PMC8407431; doi:10.1128/mSystems.00396-21)

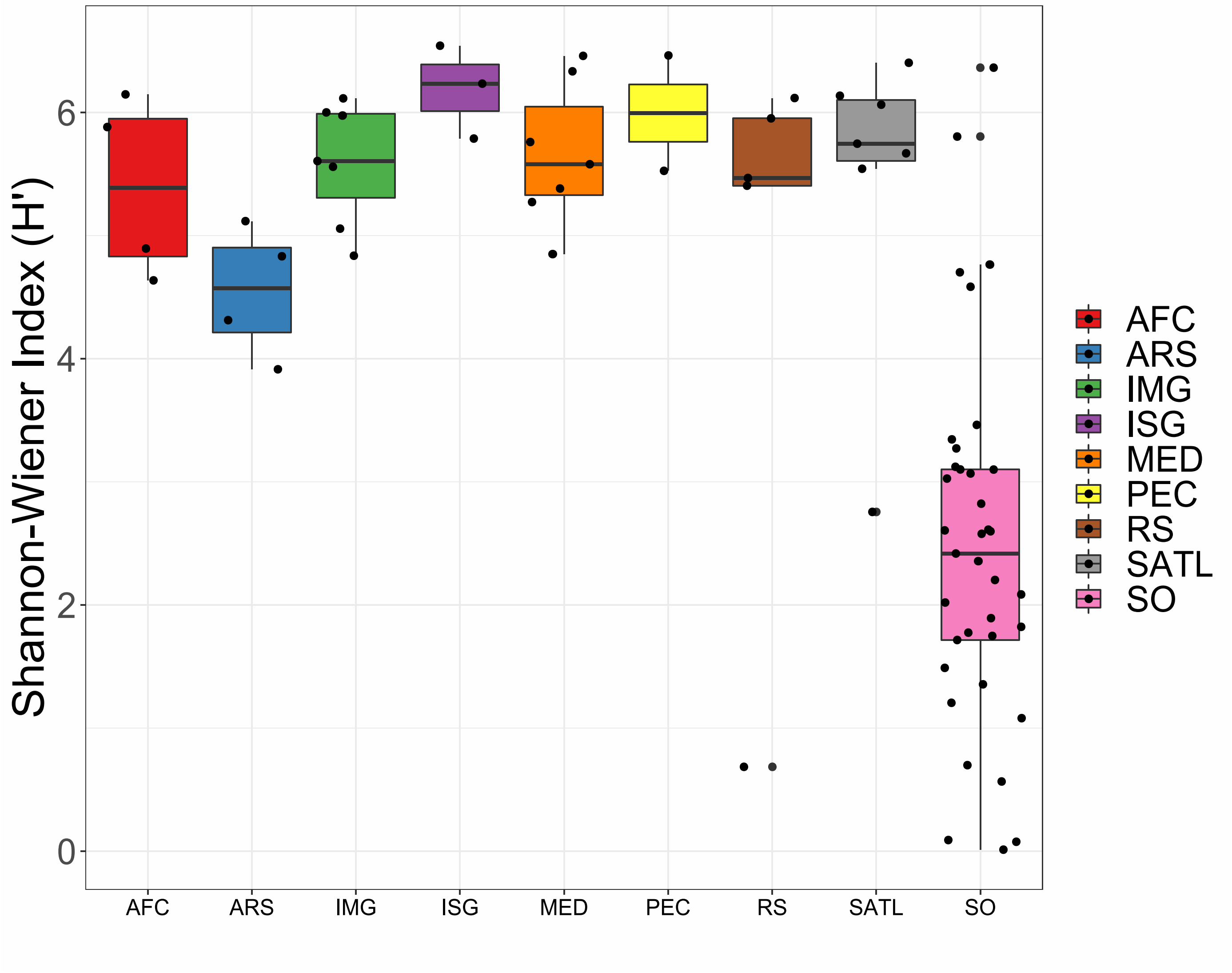

Supplement: FIG S1 [file msystems.00396-21-sf001.tif]

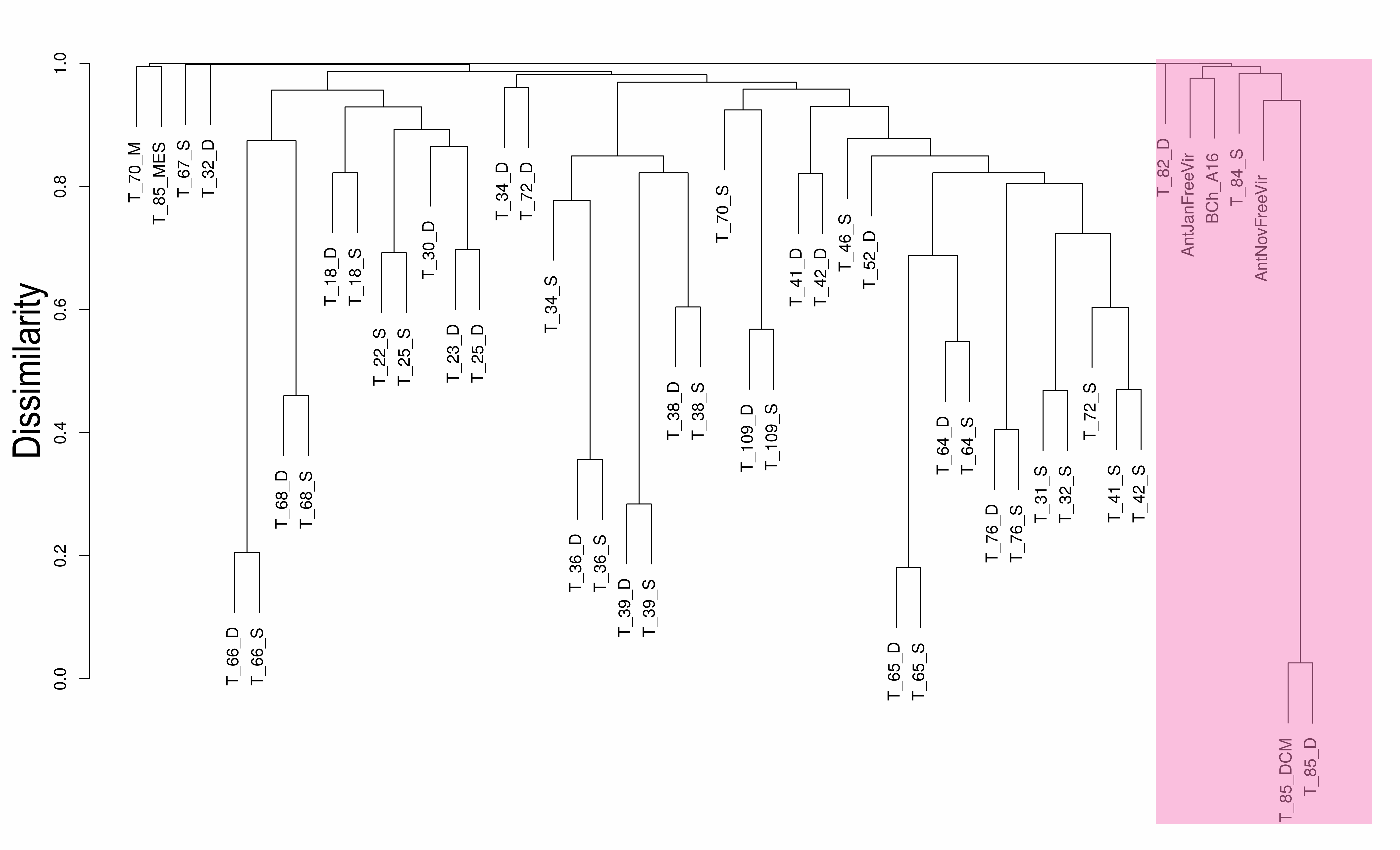

Supplement: FIG S2 [file msystems.00396-21-sf002.tif]

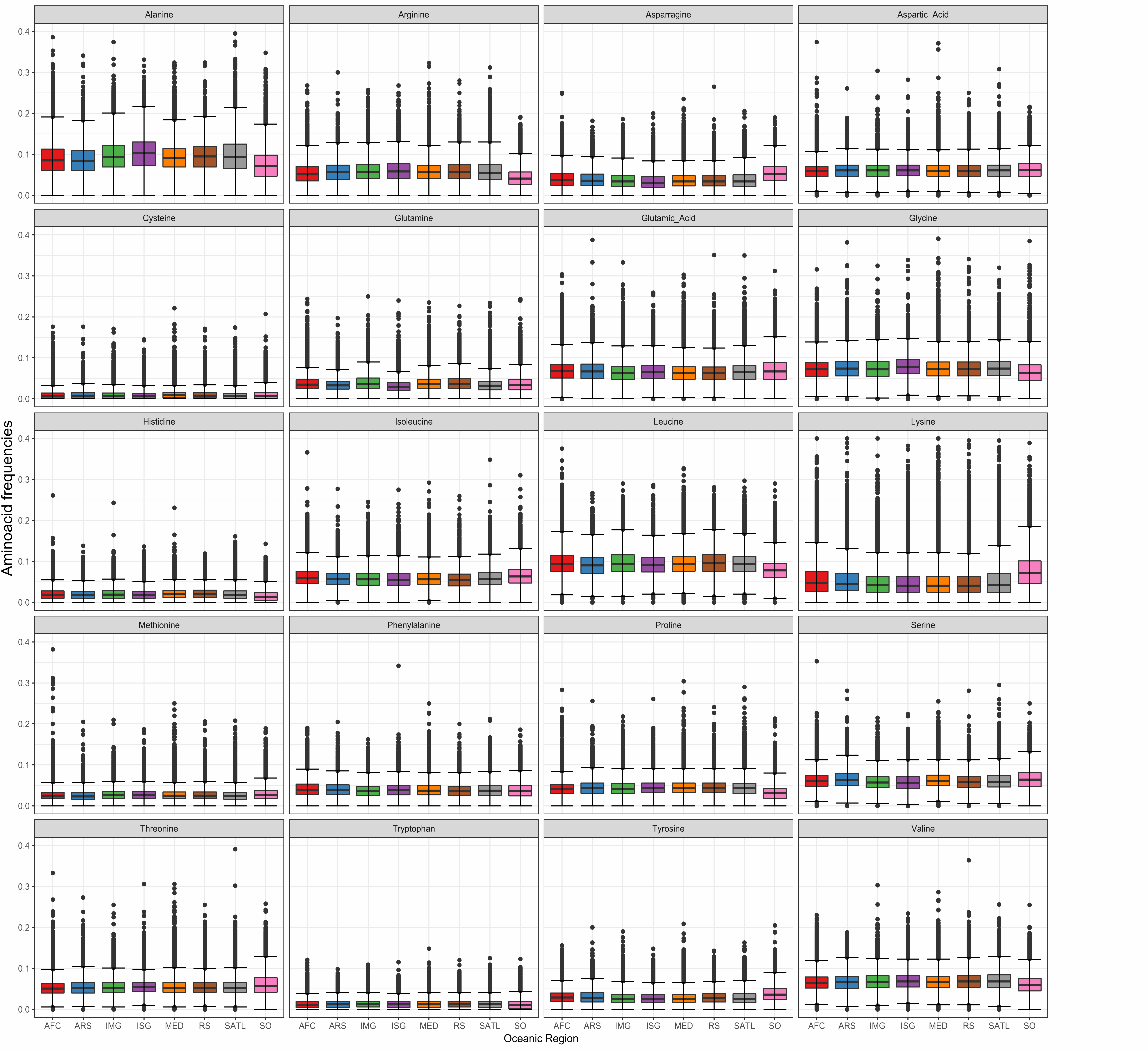

Supplement: FIG S3 [file msystems.00396-21-sf003.tif]
